# Supplementary material for: Experiences of rehabilitation one year after breast cancer diagnosis–A focus group study from the ReScreen randomized controlled trial
Source: PLoS One. 2025 Feb 13;20(2):e0315814. doi: 10.1371/journal.pone.0315814 (PMC11824999; doi:10.1371/journal.pone.0315814)
Supplement: Attachment — Submitted filename: Response to Reviewers.docx [file pone.0315814.s002.docx]

Dear Editor,

Please find attached the revised manuscript entitled ” Experiences of rehabilitation one year after breast cancer diagnosis – A focus group study from the ReScreen randomized controlled trial” (PONE-D-24-21825). We are very grateful for the reviewers´ thorough and constructive comments which have been invaluable in improving the readability of the manuscript. We have now revised the manuscript to address all points raised by the reviewers. Changes have been highlighted in the manuscript using Track Changes according to instructions, added references are marked in yellow. Below, you find a point-by-point answer to reviewer´s comments. You will also find the amended statement of funding sources, as requested, with changes marked in yellow.

Best wishes,

Ing-Marie Olsson and co-authors

**Updated funding statement**

“The ReScreen study (MM) was funded in all by external funding through governmental funding of clinical research within the Swedish National Health Service (ALF funding) (award number: 200-Project0190), FORTE ([https://forte.se/en/](https://protect.checkpoint.com/v2/___https://forte.se/en/___.YzJlOnJlZ2lvbnNrYW5lOmM6bzo3Mzk4ZjU2NTE3ZGRiODYyNzE1YWE4NjRjYTQ5YTMxNDo2OjFmMGI6MzE2M2VhNjQ5NTI1NGRjNmU0MDBlY2U5OTg4NzA5NjkyYmEyMTc3NjNhMDAzNmM5ODNmZjdiNWEyN2FmMzcwODpwOlQ6Tg)) (award number: 2020-00105), the Swedish Breast Cancer Association ([https://brostcancerforbundet.se](https://protect.checkpoint.com/v2/___https://brostcancerforbundet.se___.YzJlOnJlZ2lvbnNrYW5lOmM6bzo3Mzk4ZjU2NTE3ZGRiODYyNzE1YWE4NjRjYTQ5YTMxNDo2OjQzODE6NWQzYWU1ZWI4OGQzNmIzZmUxYjFkNDczNWVhYzVkYTY0NzA2MzBkYWZjMmNkYWQ0YWNiZjkwNjI1YmEzODc1MjpwOlQ6Tg)), the Cancer and Allergy Foundation ([https://cancerochallergifonden.se](https://protect.checkpoint.com/v2/___https://cancerochallergifonden.se___.YzJlOnJlZ2lvbnNrYW5lOmM6bzo3Mzk4ZjU2NTE3ZGRiODYyNzE1YWE4NjRjYTQ5YTMxNDo2OjE0NjE6MWViMzkzYzQ3YjM3MmQ2NTE2M2E2MzNjOTE4YjExYmE1ZmFhMmVlNGE5NmRkYWI0MTlhOTdhMTczZjdhNDIzMTpwOlQ6Tg)) (award number: 220) and The Swedish Cancer Society ([https://www.cancerfonden.se/om-oss/about](https://protect.checkpoint.com/v2/___https://www.cancerfonden.se/om-oss/about___.YzJlOnJlZ2lvbnNrYW5lOmM6bzo3Mzk4ZjU2NTE3ZGRiODYyNzE1YWE4NjRjYTQ5YTMxNDo2OjMzOTA6MDhjMGVlZTZhMTA5ZTUzOTc0MTdhZWYyN2EwZjlkZjA3Nzk0YzE4MTYyNjdhOWEwYTcwNzQwMjBmOTQ4YzE5MzpwOlQ6Tg)) (award number: 230656FE). The funding bodies had no role in any components, from study design, to submission for publication. There was no additional external funding received for this study. For the purpose of Open Access, the author have applied a CC BY public copyright license to any Author Accepted Manuscript (AAM) version arising from this submission.”

**Additional Editor Comments:**

This qualitative study evaluated in a very accurate and well carried on plan of qualitative analysis the experience with Breast Cancer of women already participating in a Randomized controlled trial (RESCREEN). The described relation between the experimental study and the qualitative study design is, in my opinion, very original and interesting. The analysis considered the two main categories and four sub-categories offering an informative interpretation of the findings. The relevance of a proactive individual-based action is shown as a plus in the women perception of the health care. I appreciated the paper clear presentation. However, I suggest considering the comments by the two reviewers preparing the second version of the paper. The non-participation rates, as suggested by a reviewer should be better discussed and possibly interpreted in relation to the distress fo the women. As the other reviewer suggested, in breast cancer care the issue of body images should be more considered and experience reported.

***Answer:*** We sincerely thank the editor for the positive valuation of our study. We have adjusted our manuscript and developed the important areas suggested.

**Reviewer 1**

The results of the focus groups describe and underline very important themes in the field of oncological rehabilitation.

The study contributes to bringing evidence on the importance of developing clinical practice towards a rehabilitation that is not only global and multidimensional, but above all guided and individualised.

It seems to me that the need to combine validated screening tools, such as the DT, with a proactive and orienting intervention, which in clinical practice requires a reference figure for patients, has been well underlined.

***Answer:*** We thank the reviewer for their positive appraisal of the manuscript.

**Reviewer 1:** Half of the eligible patients involved in the RCT participate in the focus groups. What reflections can be made on this with respect to the topic of patients' needs?

***Answer:*** Thank you for pointing at this important matter. We have further developed the reasoning in the discussion section, see page 22-23, lines 508-513.

**Reviewer 1**: I would better specify the exclusion criterion for patients with cognitive impairment, as it is a rather frequent problem in people undergoing oncology rehabilitation.

***Answer:*** Thank you. Those inclusion and exclusion criteria were for the ReScreen RCT, in which all the patients in the present study had participated. That inclusion took place before the start of treatment. Therefore, patients who experienced cognitive impairment as a result of treatment were included in the ReScreen RCT and their needs were addressed in the intervention. At the inclusion into the present focus group study, the only inclusion criteria were that the participants had fulfilled their participation in the RCT, and that at most two years have passed since the beginning of treatment. No questions related to the former (i.e. ReScreen RCT) criteria were asked about. We have now clarified this in the manuscript (page 8, lines 183-185).

**Reviewer 1.** I would better describe the decision support tool referred to in line 135.

***Answer:*** Thank you for pointing this out, the decision support tool is essential for the ReScreen intervention which the women in the IG had received. We have now outlined the steps that the decision support tool entailed (page 7, lines 146-149) and clarified that more detailed information about the decision support tool can be found in the ReScreen RCT protocol (page 7, lines 154-155).

**Reviewer 1:** For clarity, I would place the interview questions in the article rather than in separate material as supporting information.

***Answer:*** Thank you, we have now included a table containing the semi-structured interview guide in the manuscript (Table 2, page 10).

**Reviewer 2**

Dear authors,
thank you for your contribution.

***Answer:*** We thank the reviewer for their comments on the manuscript.

**Reviewer 2:** Introduction: you just cited body image within your focus of interest. However, this topic has not been explored. Please, add further details about it, knowing its impact on Quality of Life in cancer patients. See: Sebri et al., 2023; Sebri; Durosini, Pravettoni, 2023.

***Answer:*** Thank you for the comment and the suggested references. One of the consequences of breast cancer treatment is indeed negative body image. In the Background, we expanded on the topic to illuminate its importance for the affected women with breast cancer (page 4, lines 77-81). Still, the focus of our study was on women’s experiences of rehabilitation and not on their experiences of consequences from diagnosis and treatment, nor specific consequences potentially affecting quality of life in this situation. Body image was not discussed during the interviews, and it was only briefly mentioned by participants. Therefore, within this study we cannot draw any conclusions about body image. This is now clarified in the discussion (page 22, lines 489-492).

**Reviewer 2:** Secondo, please, provide information regarding previous psyhcological interventions. See: Matchim et al., 2011; Mifsud et al., 2021.

***Answer:*** Thank you. This is a topic described briefly in page 4, lines 91-92. Within this study we describe the need for tailored support, and navigation in health care system. That of course include psychological interventions, and in the background, we give a few examples, as well as examples for physical interventions. We have now increased this section with examples of psychological interventions which have had promising results on wellbeing among women with BC (page 4, lines 92-94).

**Reviewer 2:** Third, please structure your hypothesis and goals in a well-done way.

***Answer:*** Thank you. In this qualitative study our aim was to explore the experience of rehabilitation after participating in an RCT. We aimed to generate insights and did not aim to test predefined hypotheses. The variation between the groups was a finding in this process of analysis, not a predefined hypothesis or goal, in line with the qualitative research design^1^.

**Reviewer 2:** Data analysis: please, provide more information about what type of analysis has been conducted in line with literature. Have you followed Braun and Clarke, for example? Literature has to be provided.

***Answer:*** Thank you for pointing this out. This has been clarified (page 11, lines 221-222).

^1^Polit, D.F., & Beck, C.T. (2017). Essentials of Nursing Research – Appraising evidence for nursing practice (9^th^ ed.). Wolters Kluwer.
